# Description and toolkit for a research participant referral service

**DOI:** 10.1017/cts.2025.33

**Published:** 2025-02-19

**Authors:** Nicholas Eberlein, Michael D. Musty, Jamie Roberts, Sierra Lindo, W. Schuyler Jones, Ranee Chatterjee

**Affiliations:** 1 Duke University Clinical and Translational Science Institute, Durham, NC, USA; 2 Duke Office of Clinical Research, Durham, NC, USA; 3 Duke Cancer Institute, Durham, NC, USA; 4 Duke University, Department of Medicine, Durham, NC, USA; 5 Duke Clinical Research Institute, Durham, NC, USA

**Keywords:** Research, participant, engagement, concierge, navigator

## Abstract

The success of clinical research studies depends on effective recruitment and retention of study participants, yet only a small fraction of patients engage in research studies, even in academic health systems. Increasing awareness of research opportunities and facilitating connections with clinical research study teams would help to improve the success of research programs. In this Special Communications, we describe the creation and evolution of and tools used for the My Research Partners Concierge Service (MRPcs) of an academic health system. The MRPcs provides a centralized point of contact or hub for patients and community members, as well as clinical research organizations and academic partners, who have research-related questions or interests. The MRPcs helps to connect the users of the service with relevant research study teams, personnel, or resources to facilitate their engagement in a clinical research program. Our experience with the MRPcs informs our recommendation that peer institutions organize similar research service hubs for their clinical research programs to help increase awareness of and participation in clinical research by the public and to help increase the success of research programs at fulfilling their ultimate goal of improving the health of their population.

## Introduction

Prospective clinical studies, particularly randomized controlled trials, are critical to advance public health. The success of clinical research studies depends on successful recruitment of participants; unfortunately; however, many studies fail to meet enrollment goals [1,2]. Less than 5% of patients participate in clinical research despite a sizable majority of U.S. adults who express willingness [3–5]. The disparity between willingness to participate and actual participation is primarily due to patients not being approached about and poor awareness of research opportunities [5,6].

Before 2018, clinical research information at our institution was disseminated by individual study teams using advertisements in clinical or public settings. The Duke Health website had a page to list research studies, but it was manually and inefficiently updated, and information could easily become outdated; so individuals interested in research opportunities typically needed to be highly motivated and make extensive efforts to find the information they needed. Most Duke Health employees are not involved in research, and the primary consultation and referral number for the health system is staffed by non-research employees. At best, callers were referred to the relevant clinical departments, but they still often faced challenges to get accurate research information due to health system employees’ knowledge gap related to research-related information.

To address this, the My Research Partners Concierge Service (MRPcs) was established in July 2017 within the Duke University Clinical and Translational Science Institute (CTSI). MRPcs utilizes a dedicated phone number and email inbox to field queries about clinical research at Duke. It serves as a fixed point of contact for anyone interested in research opportunities and connects them with the appropriate research staff. MRPcs staff are part of CTSI and serve the broader institution, which helps bridge the gap in our decentralized research governance model (Duke clinical research comprises more than two dozen research management teams, called Clinical Research Units [CRUs], who oversee the conduct and execution of clinical research).

Additionally, our institution revamped its online research listings. With the implementation of our clinical trials management system (CTMS), OnCore, we now offer current and accurate information about clinical research. The research directory uses OnCore as its primary data source, which ensures up-to-date information (like enrollment status) through daily communication with the CTMS.

In this article, we review similar services at peer institutions and describe the MRPcs – its services, utility, challenges, and future directions to facilitate participation in clinical research.

## Literature search

We performed a literature search (Table 1) to identify publications discussing comparable services at other institutions. Our search strategy included terms to identify patients engaged in research with direct institutional support in navigating available clinical trials. Our search was not intended to be an exhaustive or systematic review, but we did include terms that we felt would yield descriptions of similar types of research-related services as MRPcs. However, this search yielded no papers describing similar services to the MRPcs. The literature did reveal papers written about experiences and best practices with patient navigation with regard to clinical care. One study did describe a research concierge service, but this service was intended for researchers and not potential research participants[7]. The literature that discusses patient navigation services at peer institutions deals with navigation teams that serve a particular demographic of patients (e.g., low health literacy patients or patients with HIV) rather than adopting a more universal focus [8–10]. We did find references regarding ResearchMatch, a national online platform that electronically matches potential study participants with research studies, which is similar to the goal of MRPcs [11,12]. The key difference lies in ResearchMatch’s electronic, self-service model versus the MRPcs’ human-centered, personalized approach.

**Table 1.** Literature search terms for services similar to the my research partners concierge service (MRPcs)

#	Query	Results from 04 Oct 2024
	**Ovid MEDLINE(R) ALL<1946 to October 4, 2024>**	
1	((patient or patients or research or researcher or subject or subjects or participant or participants) adj1^#^ (navigator or navigation or concierge or “high touch” or “call center” or hotline)).ti,ab.^&^	1,675
2	((patient or patients or research or researcher or subject or subjects or participant or participants) adj1 (navigator or concierge or “high touch” or “call center” or hotline)).ti,ab.	510
3	((patient or patients or research or researcher or subject or subjects or participant or participants) adj1 (navigator or navigators or ambassador* or “touch point” or liaison* or “go between” or go-between or concierge or “high touch” or “call center*” or hotline* or “help desk*”)).ti,ab.	1027
4	3 not 2	517
	**Web of Science: Science Conference Proceedings**	
	(patient or patients or research or researcher or subject or subjects or participant or participants) AND (navigator or navigators or ambassador* or “touch point” or liaison* or “go between” or go-between or concierge or “high touch” or hotline*)	2700

# adj= adjective; ^&^ ti,ab = title, abstract.

## Landscape review

In addition to the literature search, we searched for public directories and/or websites of 63 US peer institutions within the larger framework of the Clinical and Translational Science Awards (CTSA) Program (Table 2). At the time of our search, all institutions maintained an online public-facing clinical research study directory. Fifty-six institutions listed their studies directly on their websites (including two sites dedicated solely to cancer trials), while five provided links redirecting to their study listings on ClinicalTrials.gov. Among these institutions, twenty-five sites offered general research contact information for prospective participants. Specifically, ten provided both email and phone contact details, three offered email contact only, nine provided phone contact only, and three utilized a webform exclusively.

**Table 2.** Landscape review of institutes with public-facing listing of current clinical research studies (N = 63)

Institutes	Institution general research contact	Institution general research contact information type
All 63 institutes had a public-facing listing of current clinical research studies	25 had general research contact information listed	Email only: 3 Phone only: 9
56: all studies were listed on their institutional webpages 2: cancer trials only listed on their institutional webpage 5: linked out to clinicaltrials.gov listing		Webform only: 3 Both email/phone: 10

## Scope of MRPcs service

The MRPcs serves as a dedicated point of contact that both the general public and established or potential academic/industry partners can utilize for research information at Duke. The service is designed to handle any and all queries related to clinical research. The most common users of and types of questions for the MRPcs are as follows:

- research candidates asking about a particular clinical trial or research study;
- research candidates asking about general clinical trials opportunities relevant to their circumstances (e.g., someone with a new diagnosis of muscular dystrophy);
- research candidates asking about how clinical trials or research studies work and what their rights are should they choose to participate;
- current or past Duke research participants needing to reach out to their study team;
- research sponsors, contract research organizations (CROs), or peer institutions asking about Duke’s research capabilities or interest in joining a research program;
- non-Duke medical providers asking about how to refer a patient to a clinical trial or for assessment to join a clinical trial.

**Figure 1.**
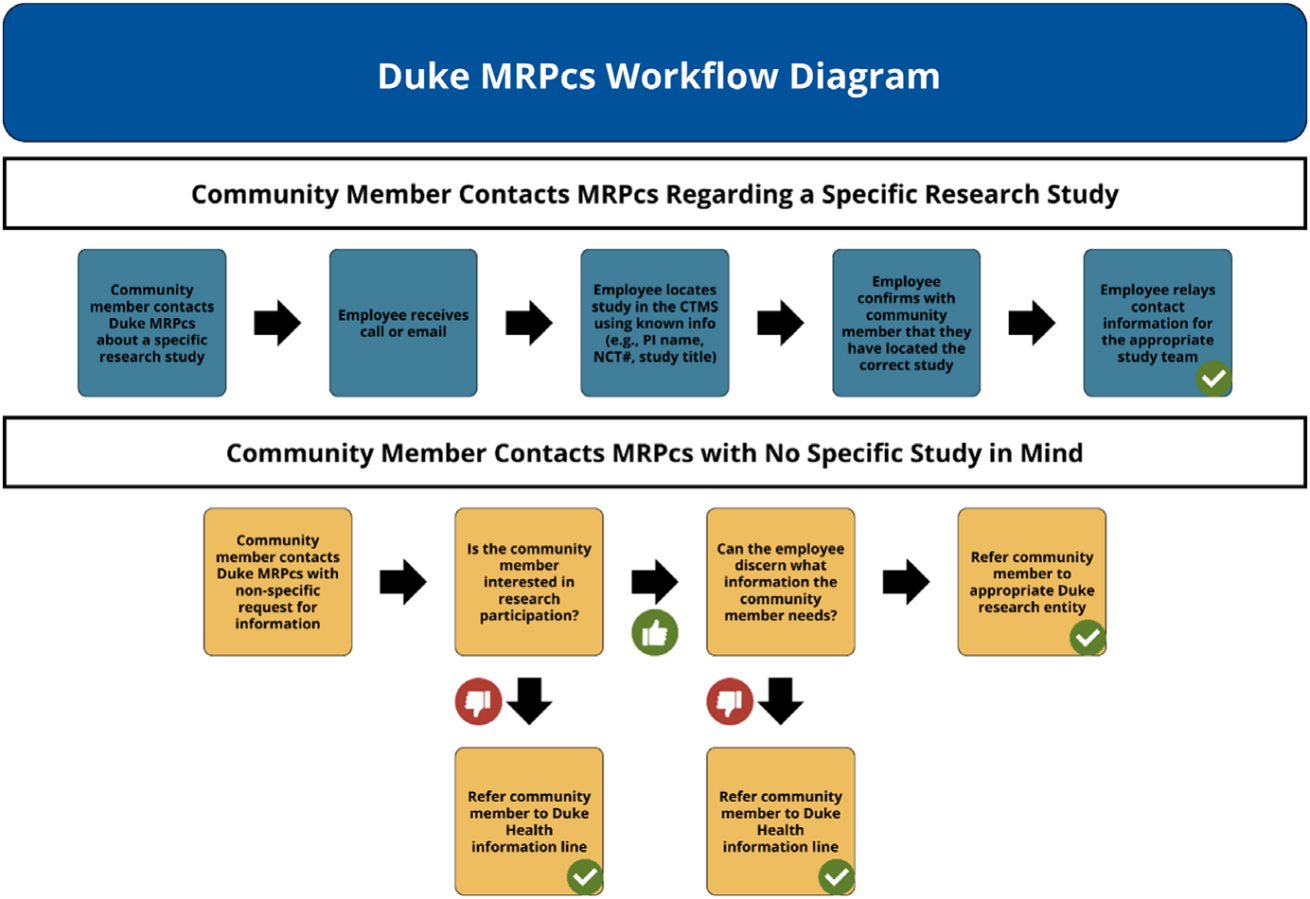
Workflows for common my research partners concierge service (MRPcs) scenarios.

The workflows for most scenarios handled by the MRPcs are depicted in Figure 1 and are described as follows. When a phone call or email comes into the MRPcs, the attendant staff member will discern the nature of the contact (e.g., perhaps it is a nurse from a non-Duke facility who wants to know if any clinical trials are available for their patient) and proceed accordingly. Following the framework of the bullet points in the preceding paragraph, most calls or emails to the MRPcs fall within six general schema and can be resolved following a structured strategy to manage each one. These strategies are described in detail in Table 3.

**Table 3.** My research partners concierge service (MRPcs) processes for different categories of queries to the MRPcs

1. Someone is interested in joining a specific clinical trial or research study.	Locate the trial/study in Duke’s institutional clinical trials management system (CTMS) and refer the interested party to the primary study coordinator.
2. Someone wants to know if there is any ongoing research that might be pertinent to them.	Seek the minimum amount of information necessary to determine the relevant medical condition(s) for research participation and refer the interested party to a known point of contact for whatever research division is appropriate for their circumstances.
3. Someone has heard about research at Duke and wants details about what participation entails and what might be available (e.g., is participation billable to their insurance, is compensation offered, how long must they participate, can they participate at their local clinic and just have drug shipped to them, does the institution have clinical trials exploring cannabis or psychedelic drugs, etc).	Answer the interested party’s questions in an objective manner according to knowledge of good clinical practice, institutional policy, and local, state, and federal regulations. If a question can only be answered subjectively, defer answering the question and provide the known point of contact for the research division that is appropriate to field subjective questions about research.
4. Someone who is enrolled in a research study or previously participated in a research study needs to contact the research team but has lost the relevant team member(s) contact information.	Seek the minimum amount of information necessary to find this person’s consent date in Duke’s clinical trials management system. Generally, asking for a first and last name is all that’s necessary. If someone shares a name with another research candidate, MRPcs staff can ask for the name or nature of the relevant study to find out which consented research participant is the correct option since study titles are attached to participants’ names and consent dates. MRPcs staff never ask for the following information: date of birth, social security number, medical records number, or home address.
5. Someone from a research sponsor, CRO, or peer institution reaches out to see if anyone at Duke might be interested in joining a research network, or if Duke is interested in granting them entry to a research network in which Duke is the central coordinating authority.	Find out the type and scope of research work relevant to the interested party and make a referral to a known point of contact for whatever research division is appropriate for their circumstances.
6. Someone from a healthcare facility outside of Duke wants to refer a patient for a clinical trial or wants to know if a clinical trial relevant to their patient’s diagnosis is available.	Seek the minimum amount of information necessary to determine the relevant medical condition(s) for research participation and refer the interested party to a known point of contact for whatever research division is appropriate for their circumstances.

Briefly, for someone interested in a particular clinical study, they can be quickly referred to the appropriate study team by locating the study in our CTMS and browsing the personnel list. Potential candidates who are not seeking a particular study but are interested in a specific therapeutic area are referred to the lead of the relevant CRU. Anyone who is curious about research regulations and their conduct can be informed by MRPcs staff members or referred to the Duke University Health System Institutional Review Board. MRPcs can search consent forms or study enrollment logs saved in our CTMS to refer current research participants who have lost a study team’s contact information back to the study team. Academic or industry partners, or referring providers outside of Duke, can be easily referred to the lead of the appropriate CRU that is relevant to their clinical research interest.

## General usage pattern

The MRPcs started in 2017. The service was introduced to the leads of Duke CRUs in their regular meetings. The service was launched over time with the inclusion of the MRPcs email address and phone number on different Duke Health platforms described below. The service was also introduced to the Duke University Health System service access staff who receive most phone calls from Duke and non-Duke patients to make them aware of the MRPcs, and they are now trained to refer patients who ask about clinical research to the service.

The MRPcs phone line and inbox were staffed at inception by the Duke CTSI employees who comprise the Recruitment Innovation Center (RIC). The MRPcs phone number and inbox are shared resources; the phone number was routed as a distinct line to the phones of each CTSI RIC team member, and each team member was able to read and reply to emails sent to the MRPcs inbox through their personal Duke Microsoft Outlook accounts. A single RIC team member was designated as the primary person to manage calls and emails made to MRPcs, and the other team members answered calls and messages when the primary person was indisposed for any number of reasons (e.g., illness, time away from the office, or a short-term need to focus more closely on other duties). All RIC team members who managed the MRPcs, either as the primary person responsible or a stand-in for the primary, had a minimum of 5 years of experience working in research at Duke.

To capture metrics and data about utilization of the service, a Research Electronic Data Capture (REDCap®) database was created that allows for the capture of basic data, including the date of contact, the nature of the query, and either the specific study staff or CRU to which the person was referred (whichever is applicable).

Utilization of the MRPcs was relatively low for the first couple of years following its inception. Staff who managed the service resolved 229 queries in 2018, which was its first full calendar year of operation. The number of queries managed by MRPcs staff more than doubled the following year, and the total number of interactions for 2019 was 590. Much of this increase between 2018 and 2019 can be attributed to specific actions initiated that year and which continue to this day:

- Duke University Health System staff who manage patient scheduling are instructed to refer or transfer people to MRPcs if anyone calls about research rather than standard care.
- The MRPcs phone number and email address are hard coded as a universal contact information on the online Duke Clinical Trials Directory (each listed study has its own contact information within its description, but the MRPcs is highlighted for people as a point of contact if they cannot find something applicable to them on the directory). The studies on the directory are listed at the discretion of each individual study team, and, until the late summer of 2024, there was no mandate that study teams must list their studies online, so the listed studies typically comprise only about 30% of all research protocols that are actively enrolling participants. For this reason, listing the contact information for MRPcs is a helpful service for studies that are enrolling but not listed on the directory.
- The MRPcs contact information is included in the research section of the patient-facing platform (Duke’s “MyChart”) of the electronic health record (EHR) “Maestro Care” (Epic Systems Corporation). The Duke University School of Medicine is a national leader in its use of leveraging patient portal recruitment messaging to introduce research opportunities to Duke University Health System patients [13]. The MRPcs contact information is included in the footer of every research message sent to individual patients, and it is also hard-coded onto the home page of the research section of the platform.

Utilization of the MRPcs spiked in 2020 with a total of 1,747 documented interactions. Of these interactions, 261 were specific to phone calls triaged from people who tested positive for COVID-19 and were seeking a clinical trial. The CTSI staff who manage the MRPcs were tasked by the Duke School of Medicine with prescreening people who called Duke following a positive COVID-19 test during the first year of the COVID-19 pandemic (2020). Until post-infection treatments (e.g., ©Paxlovid) for COVID-19 were approved or authorized for emergency use by the US Food and Drug Administration (FDA), Duke patients who tested positive were given care instructions by health system staff that included contacting MRPcs for clinical trials information. These instructions, including the suggestion to call MRPcs, stopped when trials ended early due to the rollout of COVID vaccines in early 2021. Since 2020, queries that utilize the MRPcs have normalized to a frequency of about 25 per week, with 1459 interactions in 2021, 1048 interactions in 2022, 977 in 2023, and 760 mid-way through 2024.

The therapeutic divisions to which people are most often referred by MRPcs are, in order: oncology (more than 20% of all referrals), internal medicine (includes endocrinology, rheumatology, immunology, and infectious diseases), pediatrics (includes the Duke Center for Autism and Brain Development), neurology, orthopedics, and psychiatry.

## Necessary competencies and challenges

As noted previously, the MRPcs was developed and staffed by the RIC within Duke’s CTSI. Currently, the two main individuals who manage the MRPcs are Duke employees who have both worked in various research positions at the University for more than a decade, and their job titles have included clinical research coordinator, research project planner, and research project leader. Their experience, including experience in using the clinical research systems and established relationships with the CRUs and their leaders, has been vital to the success of the MRPcs.

Proficiency in using the clinical research systems, which include the primary clinical trials management applications (©OnCore by Advarra for research management and ©Iris by iMedRIS for regulatory management) at Duke, is a critical skill to resolve any questions posed to the MRPcs. Staff members are given full read-only access to every research protocol in both applications. This allows them to answer queries related to whether a study is open to enrollment or not, who is the point of contact for any study, or if a particular study or type of study is available at Duke. We have established standard operating procedures that outline the most common questions asked and scenarios presented by people who reach out to the MRPcs, and these guidelines instruct staff on what steps should be taken to adequately resolve these queries.

In situations when someone calls to find research at Duke and there are no readily identifiable studies here, the staff member’s experience can allow them to provide other research opportunities or resources. For example, knowledge of using the ClinicalTrials.gov online database can find alternative locations for people to consider. Even if no current research opportunity is available, people can still be referred to a relevant CRU to establish a rapport for future opportunities. For example, if a person with a diagnosis of myasthenia gravis calls about a study that is now closed at Duke and all other participating sites, they can still be referred to the Neurology CRU to share their name and contact information for future studies.

The most common challenge for staff who respond to MRPcs queries is time management between MRPcs and their other job duties. The staff are committed to responding to all queries in a timely manner (immediately or within 1 business day). None of the Duke staff who interact with people on behalf of the MRPcs do so exclusively; there is simply not enough utilization of MRPcs to warrant having an employee singularly focused on this service. However, the nature of the queries, particularly when made by phone, is unpredictable, and a single interaction can take up to 30 minutes of employee time. This can interrupt an employee’s planned workflow, and repeated interruptions on particularly busy days can make it difficult to retain focus on the employee’s other tasks.

To mitigate the impact of the MRPcs on employees’ other responsibilities, the service is identified as a general information resource that is not to be used for emergent concerns. This designation gives staff the flexibility to step away from resolving queries in real time to focus on more immediate tasks and then returning later to respond to queries in the MRPcs voicemail system or inbox. Additionally, the staff who manage the MRPcs are part of the RIC within CTSI and work in close coordination with study teams across Duke to get more studies listed on Duke Health Clinical Trials Directory and provide direct contact information. These efforts increase the amount of actionable information available to the public regarding research at Duke and reduce the need to leverage MRPcs for information.

The service at Duke is currently staffed by two individuals who manage the service alongside their other tasks, requiring flexibility to handle about 25 contacts per week. A third staff member fluent in Spanish was available on demand to provide support as needed. Approximately ten percent effort for each of the two primary individuals managing the service for this volume has allowed them to meet current demand and provide coverage for each other. Weekend coverage was not provided, but voicemail and emails were followed up on Mondays. For institutions of similar size and scope, staffing may need to be scaled proportionately based on call volume. If call volume increases significantly (e.g., due to expanded outreach or new campaigns), a higher percentage of staff effort or additional personnel would be necessary.

Diversity among MRPcs staff can positively influence the service, as it might make community members from underrepresented backgrounds feel more comfortable reaching out. It could also enhance cultural competence in addressing queries. Moreover, linguistic diversity in MRPcs staff would likely increase engagement from non-English-speaking communities.

## Maintaining the service as a permanent resource

The MRPcs has proven itself to be a valuable commodity for Duke’s research program, but it does require consistent networking with staff across our broader research enterprise. Governance of clinical research at Duke is not centralized, it is divided among twenty-four CRUs with different therapeutic foci. Since this is the case, when there is a change in management personnel for any individual CRU, MRPcs staff members must communicate to the new manager(s) how general research referrals will be made to their particular CRU. In some cases, a CRU will have a shared email inbox or phone number that is used to field research inquiries, and in others, the referral is simply made directly to the research manager for a CRU. The key to successful referrals is directing community members interested in research to a point of contact that is responsive. For the most part, the MRPcs has maintained an appropriate list of internal and responsive points of contact for each CRU by periodically attending research management meetings for individual units or enterprise meetings that require attendance of all CRU managers.

## Limitations of the service

While we have found that the service provided by the MRPcs has been valuable with regard to the volume of users and the “qualitative” satisfaction of the users, as evidenced by the high quality and positive interactions that are experienced from the perspective of the MRPcs staff, we recognize that there may be limitations of the service that we are currently not identifying. We have tracked metrics for numbers of users as well as the types of research-related queries of the users. However, we have not tracked other impact measures, including the number of users that actually reached a research study team member, the number of users that ended up enrolling in a research study; and the satisfaction of the users with the MRPcs service.

## Future directions

We hope to improve the service in different ways. Currently, we promote the service only on selected Duke University research-related forums, including the Duke Clinical Trials Directory and the EHR patient portal for research messages. We plan to develop a communications strategy to heighten the service’s profile within the Duke University community as well as more publicly in order to increase awareness of and interest in research opportunities.

Since MRPcs-related duties are insufficient to fill any employee’s day, there are no current plans to task anyone to manage the MRPcs exclusively. However, having more employees trained to manage the MRPcs will allow staff to take time off from the service to focus on other job duties and to handle the potential increase in volume of users of the MRPcs that may result with increased promotion of the service. Having more language diversity among staff might help to encourage an increase in diversity of MRPcs users and ultimately research participants.

Additionally, to lessen labor burden in the future, we anticipate deploying artificial intelligence (AI) chatbots to our public-facing clinical research page. The proposed interface would be simple – community members would answer a short series of questions to be matched to clinical trials for which they might be eligible to join that are listed on the public directory (e.g., what is their age, gender, or medical diagnosis). If a match to an individual study or trial cannot be made, then people will receive the contact information for the CRU relevant to their medical diagnosis of relevance. Recent literature from 2024 provides evidence that AI interfaces can bolster recruitment from the participant user end [14]. The “patient-to-trial” matching scheme that utilizes open-source AI tools was found to be more than 90% accurate in matching patients to relevant clinical trials based on patient eligibility criteria inputs. We are confident that we can fully inform a language model with eligibility criteria for studies here in such a way that it can reliably function as a first-line referral source for patients seeking clinical trial or research study opportunities. The more nuanced and granular aspects of eligibility (e.g., medical laboratory values, comorbidities, or family medical history) that are inherent in most research protocols can be subsequently addressed and evaluated by research staff members after AI tools have been used to refer a study candidate to them. Of course, people will always have the option to abandon the AI interface in favor of calling or emailing the MRPcs staff members if they find dealing with AI confusing and/or they just prefer human-to-human interactions.

Finally, we are currently developing methods to collect and track additional metrics including satisfaction of users of the MRPcs, “success rates” in establishing contacts between users of the service with study teams, and impact on overall enrollment in clinical research studies.

## Conclusions

The MRPcs serves as a centralized resource for potentially interested research participants and clinical research peers to use to facilitate connections with clinical research programs that are most relevant to them. Since there are a vast number of individual study teams reporting to a broad array of CRUs, a centralized public-facing resource is more logistically feasible and effective than a decentralized strategy for research engagement across the entire health enterprise. In addition, the presence of the MRPcs has proven to be a safety net to prevent the following:

- missed connections between Duke and peer institutions, CROs, and research sponsors who desire Duke’s partnership;
- loss to follow-up for study participants who have lost a study team’s contact information and/or had their contact information change;
- lost potential research candidates for study teams/research studies who either do not have their research openings publicly listed or have advertising material with outdated contact information (typically, this is due to staff turnover).

Our experience with the MRPcs informs our recommendation that peer institutions organize a similar public research service hub for clinical research. Hopefully, a greater diversity of institutions with a similar service will begin to inform best practices and improve efficacy for all in order to achieve the goal of increasing participation in clinical research.

## References

[1] Huang GD , Bull J , Johnston McKee K , et al. Clinical trials recruitment planning: a proposed framework from the clinical trials transformation initiative. Contemp Clin Trials. 2018;66:74–79. 29330082 10.1016/j.cct.2018.01.003

[2] Dobra R , Wilson G , Matthews J , et al. A systematic review to identify and collate factors influencing patient journeys through clinical trials. JRSM Open. 2023;14(6):20542704231166621. 37325779 10.1177/20542704231166621 PMC10262634

[3] Finney Rutten LJ , Blake KD , Skolnick VG , Davis T , Moser RP , Hesse BW. Data resource profile: the national cancer institute’s health information national trends survey (HINTS). Int J Epidemiol. 2020;49(1):17–j. 31038687 10.1093/ije/dyz083 PMC7124481

[4] National Cancer Institute. Clinical Trial Participation Among US Adults HINTS Briefs. 2022;48 (https://hints.cancer.gov/docs/Briefs/HINTS_Brief_48.pdf) accessed August 27, 2024.

[5] National Institutes of Health. The Need for Awareness of Clinical Research. NIH Clinical Research Trials and You. 2016. (https://www.nih.gov/health-information/nih-clinical-research-trials-you/need-awareness-clinical-research) accessed August 27, 2024.

[6] Barrett NJ , Rodriguez EM , Iachan R , et al. Factors associated with biomedical research participation within community-based samples across 3 national cancer institute-designated cancer centers. Cancer. 2020;126(5):1077–1089. 31909824 10.1002/cncr.32487 PMC7021578

[7] AlFattani A , AlFirm A , AlBedah N , et al. Enhancing research support services in health organizations by implementing a “Research concierge desk”, a case study. Front Res Metr Anal. 2024;9:1335240. 38645610 10.3389/frma.2024.1335240 PMC11026691

[8] Dolan M , Walter J , Heet R. Patient navigators. New advocacy role a good fit for HIM professionals. J AHIMA. 2010;81(10):40–42. quiz 4. 21043401

[9] Lightner JS , Moore E , Barnhart T , Rajabiun S. Cost and activity analysis of patient navigation for persons with HIV: comparing health department and health clinic delivered interventions. Health Promot Pract. 2024;15248399241245059. doi: 10.1177/15248399241245059. Epub ahead of print. 38605560

[10] Budde H , Williams GA , Scarpetti G , Kroezen M , Maier CB. What are patient navigators and how can they improve integration of care? Copenhagen (Denmark): European Observatory Policy Briefs, 2022. 35129934

[11] Harris PA , Scott KW , Lebo L , Hassan N , Lightner C , Pulley J. Research Match: a national registry to recruit volunteers for clinical research. Acad Med. 2012;87(1):66–73. 22104055 10.1097/ACM.0b013e31823ab7d2 PMC3688834

[12] Pulley JM , Jerome RN , Bernard GR , et al. Connecting the public with clinical trial options: the researchMatch trials today tool. J Clin Transl Sci. 2018;2(4):253–257. 30820361 10.1017/cts.2018.327 PMC6382290

[13] Miller HN , Lindo S , Fish LJ , et al. Describing current use, barriers, and facilitators of patient portal messaging for research recruitment: perspectives from study teams and patients at one institution. J Clin Transl Sci. 2023;7(1):e96. 37125060 10.1017/cts.2023.522 PMC10130833

[14] Jin Q , Wang Z , Floudas CS , et al. Matching patients to clinical trials with large language models. Nat Commun. 2024;15(1):9074. 39557832 10.1038/s41467-024-53081-z PMC11574183

